# Endoscopic surgery affects the gut microbiota and its metabolism in breast cancer patients

**DOI:** 10.3389/fmicb.2024.1481582

**Published:** 2025-01-07

**Authors:** Jingtai Li, Fangfang Gao, Runwei Li, Zhilin Chen, Guoping Chen, Pingming Fan, Guankui Du

**Affiliations:** ^1^The First Clinical School of Hainan Medical University, Department of Breast Surgery, The First Affiliated Hospital of Hainan Medical University, Haikou, China; ^2^Key Laboratory of Tropical Translational Medicine of Ministry of Education, School of Basic Medicine and Life Sciences, Hainan Medical University, Haikou, China; ^3^Department of Biochemistry and Molecular Biology, Hainan Medical University, Haikou, China

**Keywords:** breast cancer, endoscopic surgery, gut microbiota, metabolites, 16S rRNA

## Abstract

**Background:**

Despite the advantages of endoscopic surgery in reducing trauma and enhancing recovery for breast cancer patients, its impact on gut microbiota, which is crucial for health and estrogen metabolism, remains unclear. Further investigation is necessary to fully understand this impact and its implications.

**Materials and methods:**

Between June and December 2022, fecal samples were collected from 20 patients who underwent endoscopic surgery. The gut microbiota composition was determined using 16S rRNA sequencing, while the metabolites were analyzed through liquid chromatography-tandem mass spectrometry (LC-MS/MS). Bioinformatics and statistical analyses were employed to identify significant alterations in microbial taxa abundance and to assess intergroup differences. These analyses included t-tests for pairwise comparisons, one-way ANOVA for multiple group comparisons, and chi-square tests for categorical data analysis.

**Results:**

Endoscopic surgery in breast cancer patients subtly changed gut microbiota diversity and composition. Post-surgery, there was a reduction in Lachnospiraceae, Monoglobaceae and Firmicutes to Bacteroides ratios. Shifts in metabolites were also observed, the changed metabolites impacted pathways such as primary bile biosynthesis and Ascorbate and aldarate metabolism, with PE(PGD1/18:1(9Z)) identified as a key differential metabolite that increased post-surgery. Azasetron, tyramine glucuronide, DL-DOPA, phthalide, acetophenazine, aciclovir, creatinine bicarbonate, and 4-oxo-L-proline being associated with distinct bacterial taxa.

**Conclusion:**

Breast cancer patients undergoing endoscopic surgery experience a shift in their gut microbiota and metabolic profiles. Therefore, postoperative management, with a particular focus on the adjustment of the gut microbiota, is crucial for enhancing patient recovery and health outcomes.

## Introduction

Breast cancer is the most prevalent form of cancer and one of the leading causes of death among women (Ahmad, 2019). Currently, the primary method of treating breast cancer is through surgical mastectomy (Trayes and Cokenakes, 2021; Maughan et al., 2010). During radical breast cancer surgery, inadequate aseptic procedures or a patient’s weakened immune system may result in postoperative complications such as wound infections and delayed wound healing (Sørensen et al., 2002). In recent years, endoscopic surgery has emerged as a minimally invasive technique in breast surgery (Lee et al., 2006; Lai et al., 2016). Compared to traditional open surgery, endoscopic surgery offers the benefits of reduced trauma, faster recovery, and fewer complications (Lai et al., 2016). In particular, the application of endoscopic technology in breast cancer surgery has provided patients with more options and improved treatment outcomes (Lai et al., 2016). However, further research is needed to fully understand the health implications of endoscopic surgery.

The intestinal tract is a symbiotic environment for bacteria, and the gut microbiota plays a crucial role in maintaining overall body health (Thursby and Juge, 2017). The gut microbiota produces a vast array of metabolites that interact with the host (Thursby and Juge, 2017). Breast cancer patients typically exhibit low microbial diversity and changes in microbial composition (Plaza-Díaz et al., 2019). Specifically, breast cancer patients had been found to have elevated levels of Clostridiaceae, Calcobacterium faecalis, and Ruminalococcaceae, and lower levels of Daueriaceae and Hirschsprungiaceae (Fernández et al., 2018). The degree of deterioration in breast cancer patients was found to be negatively correlated with *Faecalibacterium prausnitzii* and interleukin-6 levels (Ma et al., 2020). Additionally, research had shown that gut microbes are involved in estrogen metabolism, which is closely linked to the development of breast cancer (Parida and Sharma, 2019). However, the impact of gut microbiota on breast cancer prognosis remains largely unknown.

Studies have shown that surgery, particularly abdominal surgery, can disrupt the balance of gut microbiota (Guyton and Alverdy, 2017). This disruption can affect postoperative recovery and the incidence of complications by altering the gut microbiota (Lederer et al., 2021). A recent study has demonstrated that traditional mastectomy surgery can alter the composition and metabolites of gut microbiota (Fan et al., 2024). Therefore, this study aims to further investigate the impact of endoscopic surgery on gut microbiota, as well as the differences between endoscopic surgery and traditional mastectomy surgery in terms of their effects on gut microbiota.

## Materials and methods

### Patients

Between June 1st, 2022 and December 1st, 2022, 20 patients with breast cancer underwent endoscopic surgery at the First Affiliated Hospital of Hainan Medical College. The patients’ ages ranged from 18 to 60 years old, with a mean age of 48.75. Their BMI ranged from 21.1 to 29.3, with an average of 23.12 (Table 1).

**Table 1:** Basic information of patients.

Age	48.75 ± 10.08
BMI	23.12 ± 2.28
Height	159.14 ± 5.84
Weight	54.52 ± 5.84
Pathological type	
Non-specific invasive cancer	10
Invasive lobular carcinoma	1
Other invasive cancers	9
Pathological grade	
Grade III	5
Grade II	13
Grade I	2
Lymph node metastasis	
Transferred	9
Not transferred	11

*N = 20.*

Fecal samples were collected from patients undergoing breast cancer endoscopic surgery prior to surgery (QJ0 group), 3 days after surgery (QJ3 group), and 7 days after surgery (QJ7 group). A total of 60 samples were collected, each weighing 5 grams. After collection, the fecal samples were rapidly frozen in liquid nitrogen and stored in a refrigerator at −80°C.

### DNA extraction and 16S rRNA sequencing

The CTAB technique was employed to extract genomic DNA from the sample, and the DNA concentration was determined using the Nanodrop 2000. The sample was appropriately diluted with sterile water to a concentration of 1 ng/μL and transferred to a centrifuge tube. The primers utilized for amplification of the 16S rDNA V4 region were 515F: GTGCCAGCMGCCGCGGTAA and 806R: GGACTACNNGGGGTATCTAAT. The TruSeqR DNA PCR Free Sample Preparation Kit was utilized for library construction, which was subsequently quantified using the Life Invitrogen Qubit 3.0 and library assay. The library was sequenced using the HiSeq2500 platform after passing the assay.

### Analysis of 16S rRNA sequencing data

All data analysis was conducted on the Majorbio platform.^1^ The Flash software was utilized to achieve bipartite sequence splicing at the paired ends. The QIIME software was employed to construct water abundance tables for each taxonomy and determine beta diversity distances. USEARCH was utilized to generate OTU statistics. GreenGenes was utilized for the annotation of the rRNA database for comparison purposes. The Wilcoxon rank sum test was utilized to determine intergroup differences. Linear discriminant analysis Effect Size (LEfSe) was employed to identify bacterial taxa with significant differences in abundance between phyla and genera (LDA >2, *p* < 0.05).

### Comparisons of fecal metabolite profiles

To elucidate distinct fecal metabolomic profiles distinguishing major depressive disorder (MDD) subjects from healthy controls (HC), gas chromatography-mass spectrometry (GC-MS; Agilent 7890A coupled with 5975C) was employed. Resultant three-dimensional data sets—comprising retention time-mass-to-charge ratio (RT-*m*/*z*) pairs, sample identifiers, and standardized peak area proportions—were subsequently imported into SIMCA-P + 14.0 software (Umetrics, Umeå, Sweden). Principal coordinates analysis (PCoA) served as a visual tool for discernibly segregating before and after surgery samples based on their metabolomic fingerprints. For each metabolite, an ROC curve was constructed using binary classification outcomes. Area under the curve (AUC) values served as quantitative measures of discriminative performance, where AUC closer to 1 indicated superior discriminatory capability.

### Statistical methods

The data was analyzed using statistical software SPSS21.0 and Excel. The measurements were presented as mean ± standard deviation. An independent samples *t*-test was employed to compare the two groups. One-way ANOVA was used to compare multiple groups. The chi-square test was used to analyze count data, with a significance level of *p* < 0.05.

## Results

### Effect of endoscopic surgery on the α-diversity of gut microbiota in breast cancer patients

**Figure 1:**
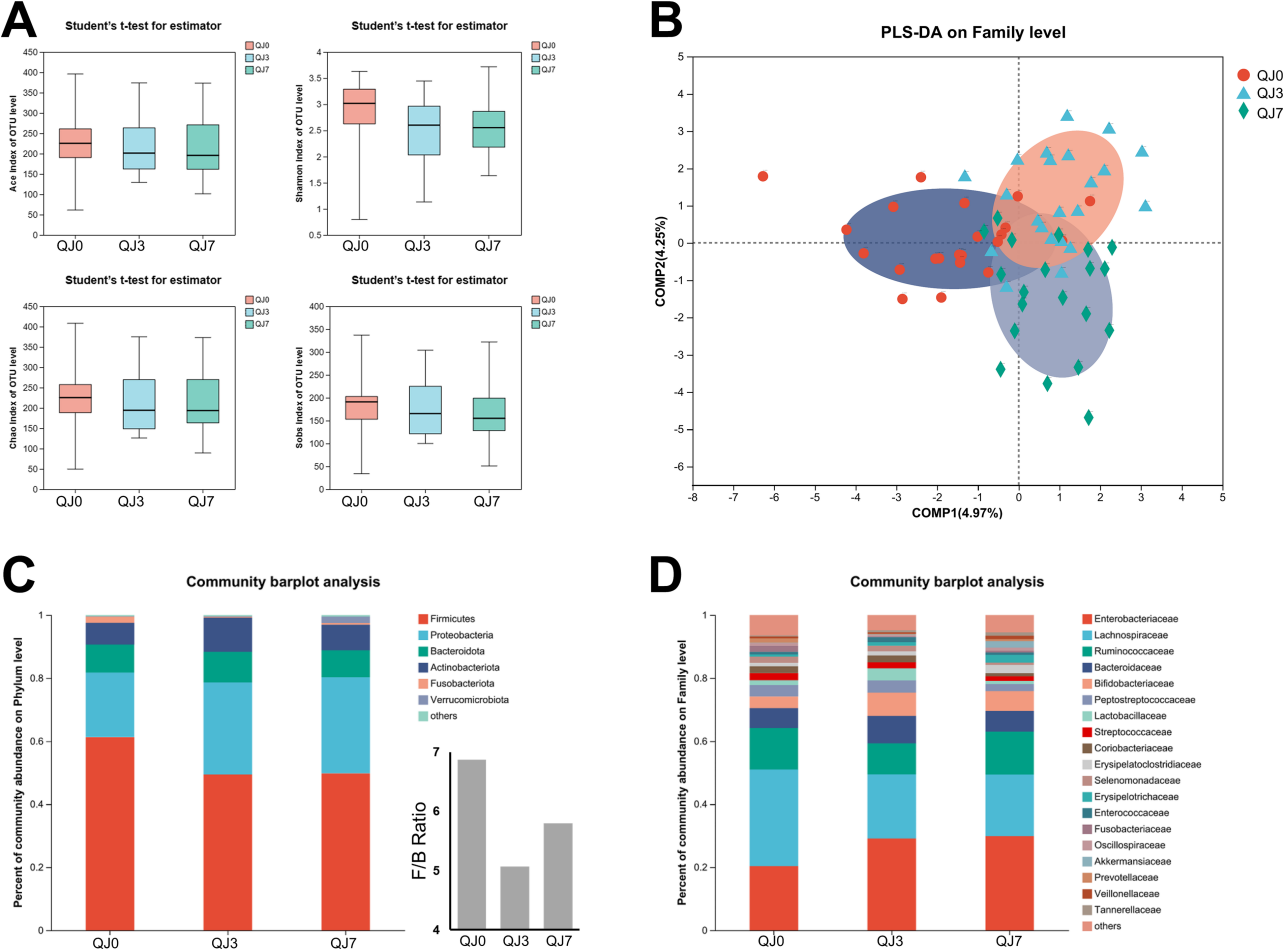
Analysis of gut microbiota diversity and composition in breast cancer patients after endoscopic surgery. **(A)** Alpha-diversity analysis. **(B)** Beta-diversity analysis. **(C)** Phylum level. **(D)** Family level.

To investigate the impact of endoscopic surgery on the gut microbiota of breast cancer patients, fecal samples were collected before and after surgery and analyzed using 16S rRNA sequencing (Figure 1A). OTU analysis revealed that the QJ0, QJ3, and QJ7 groups had 674, 597, and 631 OTUs, respectively. Alpha diversity analysis of the gut microbiota of patients 3 and 7 days after surgery showed no significant differences in the Ace index and Shannon index among groups. Furthermore, the impact of surgery on the β-diversity of gut microbiota in breast cancer patients was determined by PLS-DA analysis. The QJ0, QJ3, and QJ7 groups were slightly overlapped and distinguishable (Figure 1B).

### Effect of endoscopic surgery on the composition of gut microbiota in breast cancer patients

The composition of the gut microbial community in the patients was further investigated (Figures 1C,D). The most prevalent microbes at the phylum level were Firmicutes, Proteobacteria, Bacteroides, and Actinobacteria. Firmicutes and Proteobacteria accounted for 81.76% of the overall phylum abundance. Moreover, the families Lachnospiraceae, Enterobacteriaceae, and Ruminococcaceae were the most abundant, comprising more than 60% of the total. Additionally, the Firmicutes to Bacteroides ratios were significantly lower in the QJ3 and QJ7 groups compared to the QJ0 group.

### Analysis of differential gut microbiota

**Figure 2:**
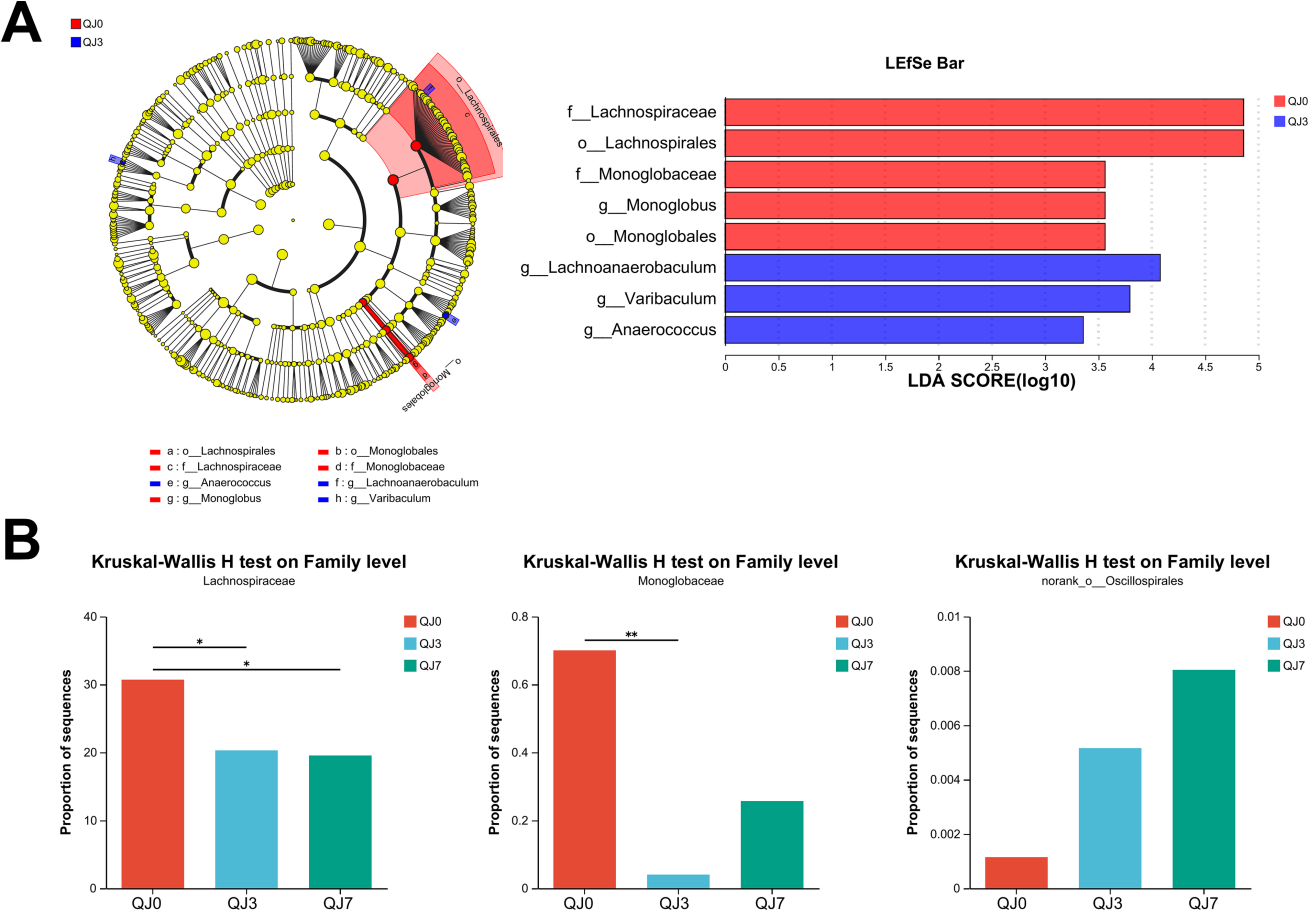
Analysis of differences in gut microbiota among patients with breast cancer after endoscopic surgery. **(A)** LEfSe multilevel species difference discriminant analysis. **(B)** Comparison of family levels among multiple groups. ^*^Indicates the difference between groups *p* < 0.05. ^**^Indicates the difference between groups *p* < 0.01.

LEfSe analysis identified three distinct bacteria, including f__Lachnospiraceae (*p*-value = 0.0271), o__Lachnospirales (*p*-value = 0.0271), and g__Lachnoanaerobaculum (*p*-value = 0.0182) (Figure 2A).

The Kruskal–Wallis *H* test analysis was used to detect bacteria with significant differences. Lachnospiraceae (*p*-value = 0.0271) was greatly reduced, while Monoglobaceae (*p*-value = 0.0457) and norank_o__Oscillospirales (*p*-value = 0.0488) were significantly enhanced (Figure 2B).

### Effect of endoscopic surgery on the metabolism of gut microbiota in patients with breast cancer

**Figure 3:**
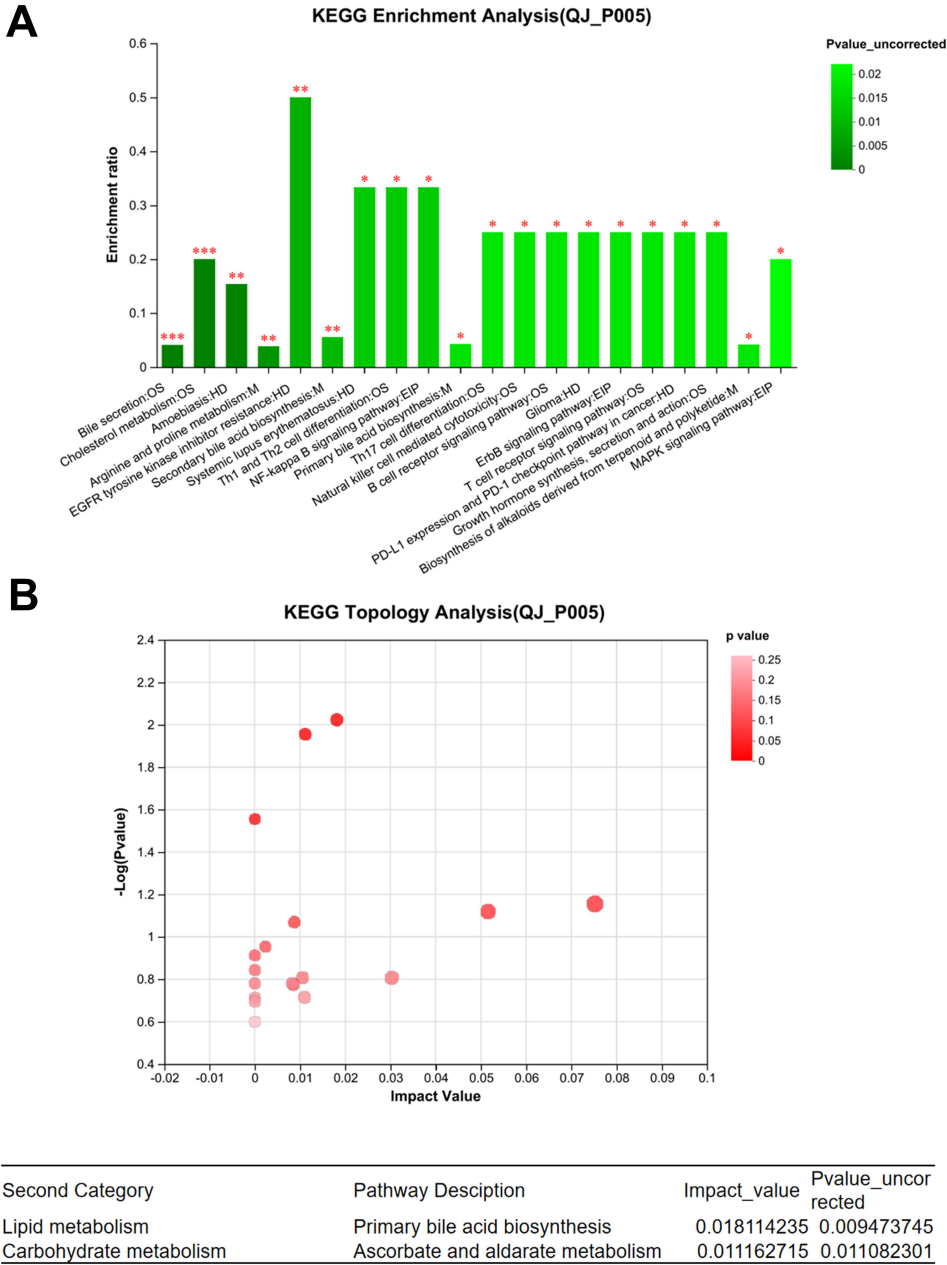
Analysis of metabolites in gut microbiota in patients with breast cancer after endoscopic surgery. **(A)** Enrichment analysis of differential metabolite KEGG functional pathways. **(B)** KEGG topology analysis. ^*^Indicates the difference between groups *p* < 0.05. ^**^Indicates the difference between groups *p* < 0.01. ^***^Indicates the difference between groups *p* < 0.001.

In the positive ion mode, a total of 85 different metabolites were identified between QJ0 and QJ3, while 153 different metabolites (*p* < 0.05) were detected between QJ0 and QJ7. In the negative ion mode, 44 and 67 different metabolites (*p* < 0.05) were found in QJ3 and QJ7, respectively, compared to QJ0. The KEGG functional pathway analysis revealed that 20 signaling pathways were significantly enriched, including bile secretion, phenylalanine metabolism, and cholesterol metabolism (Figure 3A). The KEGG topology analysis showed that primary bile acid biosynthesis and metabolism of ascorbate and aldarate were significantly affected (Figure 3B).

**Figure 4:**
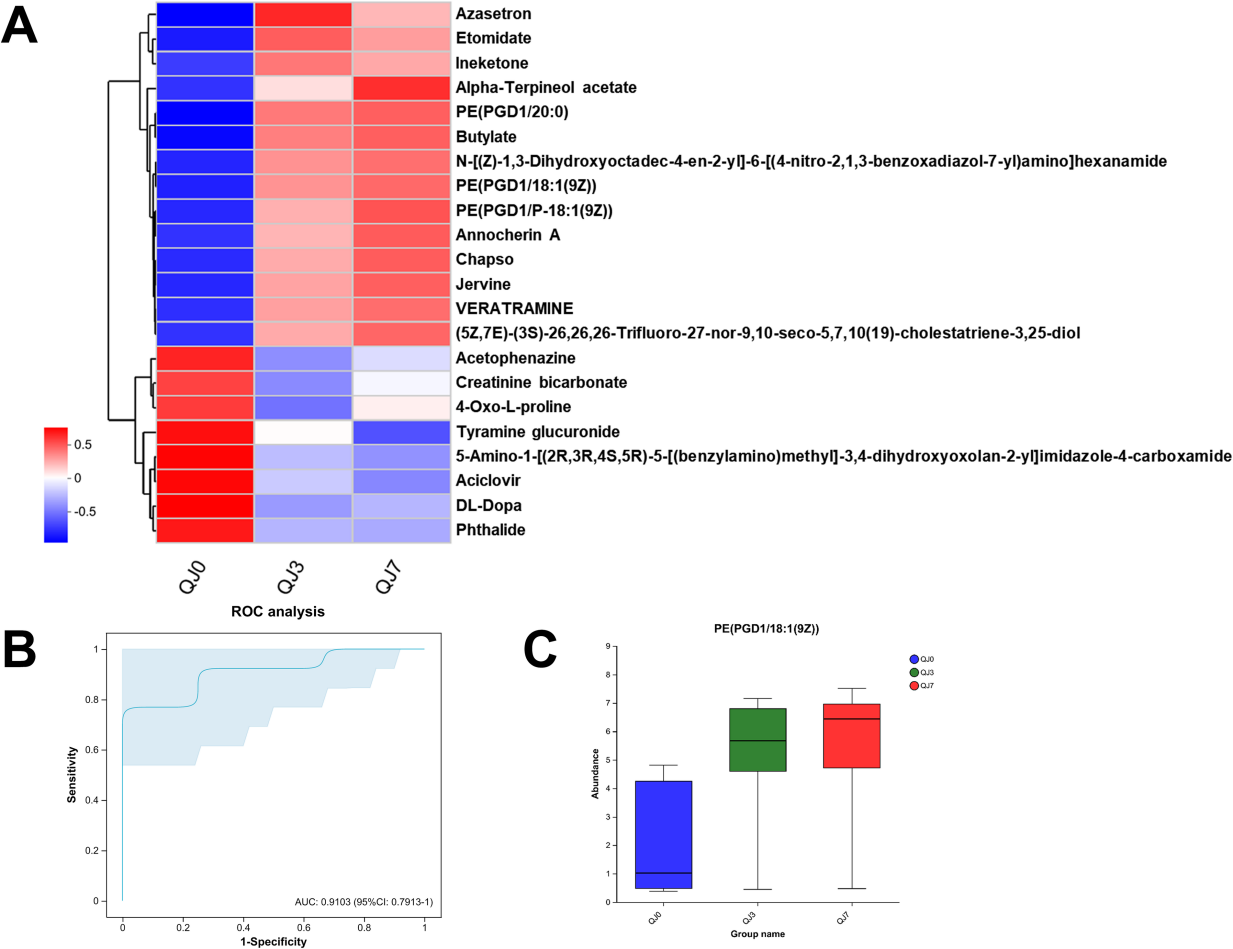
Differential metabolite analysis. **(A)** Cluster analysis of differential metabolites. ROC analysis **(B)** and abundance **(C)** of differential metabolites in breast cancer patients after endoscopic surgery.

Consequently, we adjusted the level of significant difference to *p* < 0.01, and post-hoc tests for multiple group comparisons identified a total of 22 differential metabolites. Heatmap analysis revealed that these differential metabolites could be categorized into two groups (Figure 4A). The metabolites that increased post-surgery include azasetron, etomidate, ineketone, alpha-terpineol acetate, PE(PGD1/20:0), butylate, N-[(Z)-1,3-dihydroxyoctadec-4-en-2-yl]-6-[(4-nitro-2,1,3-benzoxadiazol-7-yl)amino]hexanamide, PE(PGD1/18:1(9Z)), PE(PGD1/P-18:1(9Z)), annocherin A, chapso, jervine, veratramine, (5Z,7E)-(3S)-26,26,26-Trifluoro-27-nor-9,10-seco-5,7,10(19)-cholestatriene-3,25-diol. The metabolites that decreased post-laparoscopic surgery include acetophenazine, creatinine bicarbonate, 4-oxo-L-proline, tyramine glucuronide, 5-amino-1-[(2R,3R,4S,5R)-5-[(benzylamino)methyl]-3,4-dihydroxyoxolan-2-yl]imidazole-4-carboxamide, aciclovir, DL-DOPA, phthalide.

The ROC analysis identified an important differential metabolite, PE(PGD1/18:1(9Z)) [AUC = 0.9103, 95% CI: (0.7913–1)], whose abundance gradually increased at 3 and 7 days after surgery compared to before surgery (Figures 4B,C).

### Association study of differential metabolites with gut microbiota

**Figure 5:**
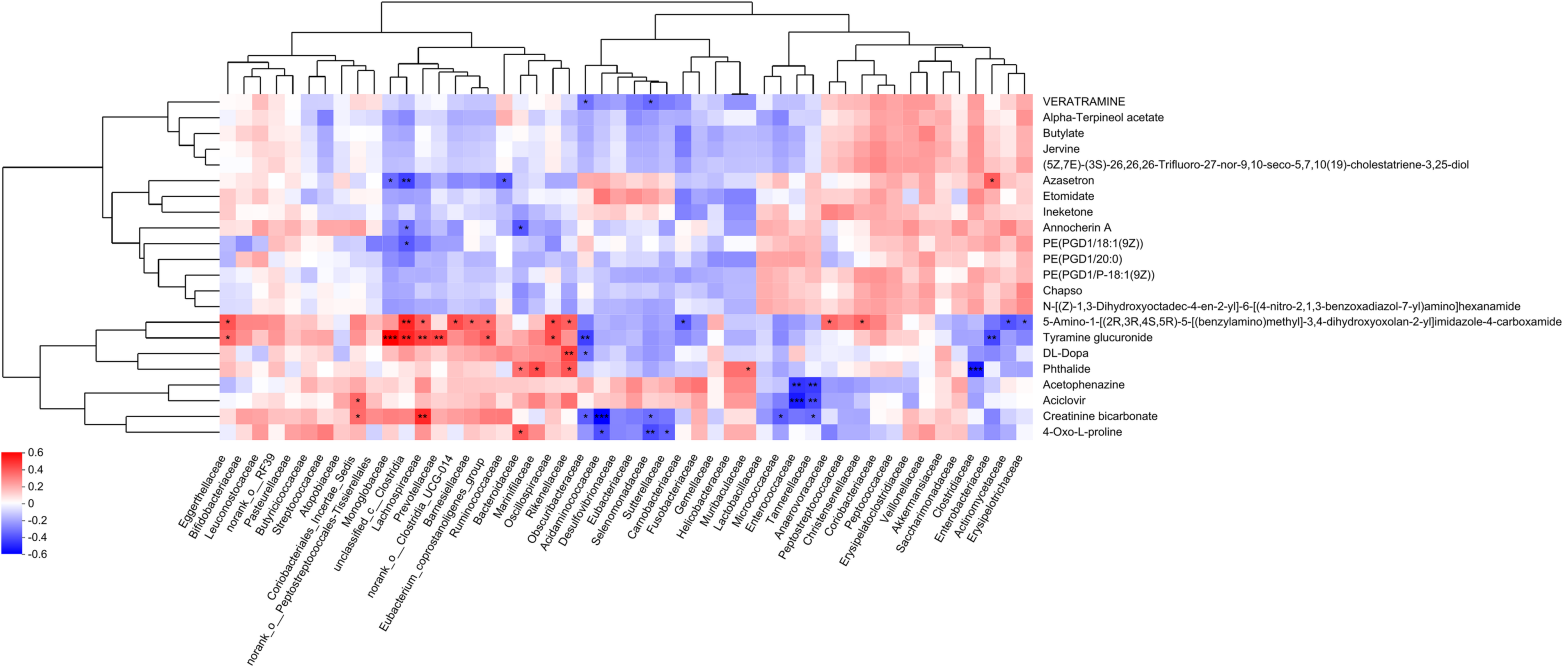
Correlation analysis between differential metabolites and microbiota. The right-hand side of the figure lists the names of the differential metabolites, while the base presents the gut microbiota. Each cell within the matrix represents the correlation between two attributes—metabolites and associated characteristics. The varying colors within the cells denote the magnitude of the correlation coefficients between the attributes. Asterisks indicate the significance of the *p*-values, with the following designations: ^*^p < 0.05, ^**^p < 0.01, and ^***^p < 0.001.

Figure 5 delineates the correlations between 22 differentially expressed metabolites and the gut microbiota. The production of azasetron and 5-amino-1-[(2R,3R,4S,5R)-5-[(benzylamino)methyl]-3,4-dihydroxyoxolan-2-yl]imidazole-4-carboxamide is significantly correlated with the presence of unclassified_c__Clostridia. The synthesis of tyramine glucuronide is significantly associated with Monoglobaceae, unclassified_c__Clostridia, Lachnospiraceae, Prevotellaceae, Obscuribacteraceae, and Enterobacteriaceae. The generation of DL-DOPA is significantly correlated with Rikenellaceae. Phthalide production is significantly linked to Clostridiaceae. The synthesis of acetophenazine and aciclovir is significantly associated with Enterococcaceae and Tannerellaceae. Creatinine bicarbonate production is significantly correlated with Lachnospiraceae and Acidaminococcaceae. Lastly, the production of 4-oxo-L-proline is significantly associated with Selenomonadaceae (see Table 1).

## Discussion

Evidence from prior studies underscores the profound impact of surgical intervention on the intestinal ecosystem. Surgery for colorectal cancer has been found to modify the composition of bacterial groups, including bifidobacteria and lactobacilli (Zaharuddin et al., 2019). Changes in perioperative microbiota during cardiac surgery have been shown to impact prognosis (Chernevskaya et al., 2021). Following liver transplantation, the microbiota plays a crucial role in immunity and metabolism, which correlates with overall health (Ancona et al., 2021; Bromberg et al., 2015). Open surgeries for breast cancer have also been found to induce substantial alterations in the microbiota (Fan et al., 2024). Against this backdrop, our investigation specifically targets the underexplored terrain of endoscopic surgery—a minimally invasive approach—and its repercussions on the gut microbiota and metabolites.

Our current investigation solidifies the premise that endoscopic surgery exerts a notable effect on both the beta diversity and compositional profile of the gut microbiota, reaffirming surgery’s status as a potent modifier of gut ecology. Specifically, our results revealed a striking diminution in the Firmicutes to Bacteroidetes ratio among individuals undergoing endoscopic surgery for breast cancer. This finding aligns closely with observations from a study demonstrating reduced Firmicutes to Bacteroidetes ratios in breast cancer survivors versus healthy counterparts (Caleca et al., 2023), further underscoring surgery’s role in reconfiguring microbial landscapes. In a cohort with a history of chemotherapy, higher levels of fear of cancer recurrence were associated with lower microbial diversity, lower relative abundance of Firmicutes, and higher relative abundance of Bacteroidetes (Okubo et al., 2020). Adjuvant letrozole and radiotherapy resulted in a shift in the gut microbial dominance from Firmicutes to Bacteroidetes (Vilhais et al., 2024). Recent research has shown that surgery leads to a decrease in the abundance of Firmicutes and Lachnospiraceae (Fan et al., 2024). Therefore, our study enriches our comprehension of post-breast-cancer treatment microbiome alterations.

This study found that endoscopic surgery led to a significant increase in the abundance of Oscillospirales, and a reduction in the abundance of the Lachnospiraceae and Monoglobaceae. Oscillospirales are considered as potential next-generation probiotic candidates (Murtaza et al., 2024). Oscillospira is negatively correlated with obesity, obesity-related chronic inflammation, and metabolic diseases (Yang et al., 2021). The abundance of Oscillospirales is positively correlated with low body mass index and can be used as an indicator for predicting the development of childhood obesity (Chen et al., 2020). Members of the Lachnospiraceae family primarily influence the host by producing short-chain fatty acids, and converting primary bile acids into secondary bile acids (Cesic et al., 2023; Telle-Hansen et al., 2022; Borton et al., 2017). Several studies have shown that the reduction of Lachnospiraceae is associated with various conditions such as allergies, inflammatory bowel disease, and metabolic disorders (Liu et al., 2023; Thipart et al., 2023; Huang et al., 2023). Lachnospiraceae and its metabolite butyric acid have significant anti-inflammatory effects, which can regulate immune responses, reduce inflammatory reactions, and promote the differentiation of CD4^+^ T cells into regulatory T cells (Cesic et al., 2023; Sun et al., 2022). The relative abundance of Lachnospiraceae in the intestines of breast cancer patients decreased (Di Modica et al., 2021; Donati Zeppa et al., 2023). A study showed that the decline in the abundance of Monoglobaceae bacteria in the elderly may affect their immune response (Gamez-Macias et al., 2024). Therefore, after breast cancer patients have received treatment, they may need to adjust their diet or use drug intervention to affect the abundance of gut microbiota after receiving treatment.

Our research has revealed significant shifts in gut metabolites of breast cancer patients after endoscopic surgery, marked by enriched bile secretion, cholesterol metabolism, and ascorbate and aldarate metabolism. Bile acids and their derivatives have been shown to inhibit various tumor cell lines, including colon cancer, breast cancer, pancreatic cancer and leukemia (Zhu et al., 2022; Wu et al., 2022; Wang et al., 2022). Lithocholic acid (LCA) can inhibit the adipogenesis of breast cancer cells and induce apoptosis of these cells through its putative cytotoxic effects (Kovacs et al., 2019). Bile acids signal through their receptors such as farnesoid X receptor (FXR) and G-protein-coupled bile acid receptor 1 (TGR5) (Baumeister et al., 2024). FXR agonism by the bile acid mimetic known commercially as ocaliva, or obeticholic acid, significantly reduced breast cancer progression and overall tumor burden in a pre-clinical model (Joseph et al., 2024). In breast cancer, cholesterol and its metabolites have been found to promote tumor progression (Zeng et al., 2024). Hypercholesterolemia is considered a risk factor for estrogen receptor positive breast cancer and is associated with decreased tumor response to endocrine therapy (Nelson et al., 2013). In addition, obesity and altered lipid metabolism are also one of the risk factors for breast cancer in premenopausal and postmenopausal women, partly due to the effect of cholesterol on biophysical properties of cell membranes and the impact of these changes on signaling events initiated on membranes (McDonnell et al., 2014). Cholesterol metabolites such as 27-hydroxycholesterol play a particularly significant role in breast cancer. 27-hydroxycholesterol can not only promote the growth of estrogen receptor-positive breast cancer cells, but also stimulate cell proliferation and metastasis in several breast cancer models (Vini et al., 2022; DeRouen et al., 2023). Furthermore, 27-hydroxycholesterol functions as an endogenous regulator of lipid metabolism by interacting with nuclear liver X receptor (LXR) α and LXR β. It inhibits the anti-tumor immune response and recruits pro-angiogenic and immunosuppressive neutrophils, thereby promoting tumor progression (Molostvov et al., 2023; Krawczynska et al., 2024). The metabolism of ascorbate and aldarate involves multiple proteins and metabolites, which are metabolized in the cytoplasm and endoplasmic reticulum. By modulating these pathways, the level of ascorbate can be controlled, thereby influencing the growth and survival of tumor cells (Sanookpan et al., 2023). Ascorbic acid exerts its anticancer activity through two main mechanisms: induction of oxidative stress by hydrogen peroxide and DNA demethylation mediated by the activation of eleven translocase enzymes (Steers et al., 2023; Zarakowska et al., 2024). This suggests that ascorbic acid may have a role in cancer treatment through different biological pathways, including direct cytotoxic effects and indirect effects on genome stability (Zarakowska et al., 2024). Therefore, endoscopic surgery may impact the metabolic function of gut microbiota in breast cancer patients, which in turn may affect the prognosis.

## Conclusion

In conclusion, although endoscopic surgery has a certain impact on the intestinal microbial diversity of breast cancer patients, there is only a slight change in the β diversity of intestinal microorganisms before and after surgery. However, there is still a significant difference between the groups, indicating that surgery may lead to a partial change in the composition of microorganisms. After surgery, the proportion of some bacterial groups, such as Firmicutes and Bacteroidetes, changed significantly, especially in the QJ3 and QJ7 groups. Furthermore, the types and quantities of metabolites produced by patients’ gut microbes have also changed. Although the current study provides us with some preliminary understanding of the impact of endoscopic surgery on the gut microbes of breast cancer patients, the specific mechanisms and long-term effects still require further in-depth study.

## Data Availability

The datasets presented in this study can be found in online repositories. The names of the repository/repositories and accession number(s) can be found at: https://www.ncbi.nlm.nih.gov/, PRJNA1110396.

## Footnotes

^1^ https://cloud.majorbio.com
